# MIC-based tuberculosis drug susceptibility testing using Sensititre MYCOTB: a diagnostic accuracy meta-analysis

**DOI:** 10.1128/jcm.00813-26

**Published:** 2026-08-20

**Authors:** Daniel Raj D, Mukesh Kumar G, Jitendra Singh, Sabir Ali, Shaina Gaikwad, Tharani Mohankumar, Swathi S, Shashank Purwar, Debasis Biswas, Anand Kumar Maurya, Sanjit Sah, Aroop Mohanty, Rachana Mehta

**Affiliations:** 1 Medical Laboratory Technology (Microbiology), AIIMS 390706, Bhopal, Madhya Pradesh, India; 2 Psychiatric Nursing, AIIMS 519296 , Bhopal, Madhya Pradesh, India; 3 Department of Translational Medicine, AIIMS 390706, Bhopal, Madhya Pradesh, India; 4 Medical Research Unit (MRU), AIIMS 390706, Bhopal, Madhya Pradesh, India; 5 Department of Microbiology, AIIMS 390706, Bhopal, Madhya Pradesh, India; 6 Department of Paediatrics, Dr D.Y. Patil Medical College, Hospital and Research Centre. Dr. D.Y. Patil Vidyapeeth (Deemed-to-be-University) 121766 , Pune, Maharashtra, India; 7 Department of Public Health Dentistry, Dr. D.Y. Patil Dental College and Hospital, Dr. D.Y. Patil Vidyapeeth 69758, Pune, Maharashtra, India; 8 Department of Medicine, Korea University 34973, Seoul, South Korea; 9 Department of Microbiology, AIIMS 560851, Gorakhpur, Uttar Pradesh, India; 10 Clinical Microbiology, RDC, Manav Rachna International Institute of Research and Studies 231547, Faridabad, Haryana, India; 11 SR Sanjeevani hospital, Kalyanpur, Siraha, Nepal; Vanderbilt University Medical Center, Nashville, Tennessee, USA

**Keywords:** phenotypic drug susceptibility test, sensititre MYCOTB AST plate, gold standard DST, method comparison

## Abstract

Accurate drug susceptibility testing (DST) is crucial for designing effective regimens for multidrug-resistant (MDR) and pre-extensively drug-resistant tuberculosis (pre-XDR TB). Sensititre MYCOTB enables simultaneous determination of minimum inhibitory concentrations (MICs) for multiple drugs, but its diagnostic performance varies across studies. This meta-analysis evaluated the diagnostic performance of Sensititre MYCOTB for key MDR and pre-XDR TB drugs. The protocol was registered in PROSPERO (CRD420251230599). PubMed, Cochrane, Google Scholar, Scopus, ONOS, Web of Science, ScienceDirect, and registries were systematically searched for studies published between 2010 and 2025. Studies comparing the Sensititre MYCOTB with reference DST for *Mycobacterium tuberculosis* complex (MTBC) were included. Bias assessment and pooled diagnostic accuracy estimates were generated. Fourteen studies, including 1,728 isolates, were analyzed. Rifampicin and isoniazid demonstrated high sensitivity (0.976 [95% CI: 0.94–0.99] and 0.977 [95% CI: 0.95–0.99]) and specificity (0.958 [95% CI: 0.84–0.98] and 0.957 [95% CI: 0.83–0.99], respectively) with low heterogeneity. Amikacin, kanamycin, and ofloxacin demonstrate good diagnostic accuracy, with high specificity (>0.98 [95% CI]). Moderate diagnostic accuracy was observed for ethambutol, streptomycin, ethionamide, and rifabutin. Cycloserine, moxifloxacin, and para-aminosalicylic acid showed inconsistent performance despite excellent specificity (>0.97 [95% CI]). Sensitivity analysis partially improved pooled sensitivity for moxifloxacin 0.801 (95% CI: 0.585–0.924) and para-aminosalicylic acid 0.76 (95% CI: 0.518–0.894), whereas cycloserine remained at 0.436 (95% CI: 0.190–0.725), although heterogeneity persisted. Sensititre MYCOTB DST demonstrates high diagnostic accuracy for MDR-TB and pre-XDR-TB drugs, while caution is required with cycloserine, moxifloxacin, and para-aminosalicylic acid. These findings support the integration of MIC-based testing into clinical decision-making.

## INTRODUCTION

Tuberculosis (TB) remains a significant global public health concern (1), with disease control increasingly challenged by the emergence and spread of multidrug-resistant tuberculosis (MDR-TB) and pre-extensively drug-resistant tuberculosis (pre-XDR-TB). Drug-resistant TB is associated with prolonged and complex treatment regimens, increased drug-related toxicity, higher healthcare costs, and poor treatment outcomes. Early and precise drug susceptibility testing is indispensable for the comprehensive management of MDR and pre-XDR-TB to direct tailored therapy and stop further resistance amplification (2, 3).

Phenotypic DST using conventional culture-based methods, including the agar proportion method on Lowenstein–Jensen or Middlebrook media and automated liquid culture systems, particularly BACTEC MGIT 960, remains the reference standard for detecting antitubercular drug resistance. However, these methods are labor-intensive and technically demanding, with long turnaround times of up to 4–6 weeks due to the slow growth of *Mycobacterium tuberculosis* complex (MTBC) (2, 4). Variability in critical concentrations, limited standardization for second-line and newer drugs, and substantial infrastructure and labor requirements further limit their routine use, particularly in resource-limited settings (5).

The broth microdilution (BMD) method has emerged as a promising alternative to phenotypic DST, enabling the quantitative determination of minimum inhibitory concentrations (MICs). A standardized 96-well BMD platform, such as the Sensititre MYCOTB plate, allows synchronous testing of multiple first- and second-line antitubercular drugs from a single isolate. Several laboratory-based and multicenter studies have demonstrated shorter turnaround times, good reproducibility, and high categorical agreement between BMD and conventional reference methods for most drugs. Although there are several broth microdilution platforms available, we limited our research to the Sensititre MYCOTB plate to ensure methodological consistency. Direct comparability is restricted because substantial heterogeneity may be introduced by variations in drug composition, MIC ranges, and interpretation criteria among platforms, and it is the most extensively verified commercial platform (4, 6–8). CRyPTIC plates, on the other hand, are mainly used for extensive genomic and epidemiological research (9), whereas Sensititre MYCOTB is used for routine diagnostics in some clinical laboratories and for research purposes.

MIC-based DST is vital for drugs with resistance occurring along a continuum, including fluoroquinolones and newer or repurposed agents. Multiple laboratory studies emphasize the importance of validated MIC quality control ranges for reliable interpretation of BMD results (10). Although molecular DST methods enable rapid detection of resistance-associated mutations, they do not capture all resistance mechanisms or provide quantitative resistance levels, underscoring the continued importance of phenotypic MIC-based testing (11).

Despite growing evidence supporting the use of broth microdilution (BMD)-based DST, reported diagnostic performance varies across drugs and settings, with notable inconsistencies for ethambutol and some second-line agents (6–8, 12). While previous studies have evaluated broth microdilution methods, a comprehensive synthesis focused specifically on the Sensititre MYCOTB platform across multiple anti-tubercular drugs remains limited. This study provides a platform-specific and drug-level evaluation of diagnostic accuracy using hierarchical models, with emphasis on clinical interpretation in MDR and pre-XDR tuberculosis. Therefore, this review aims to evaluate the discriminatory ability of the Sensititre MYCOTB method for phenotypic DST of MTBC in MDR and pre-XDR TB by estimating pooled sensitivity, specificity, and overall diagnostic performance compared with established reference standards.

## PROTOCOL AND REGISTRATION

This review adhered to the Preferred Reporting Items for Systematic Reviews and Meta-Analyses of Diagnostic Test Accuracy Studies (PRISMA-DTA). Under registration number CRD420251230599, this review process was pre-registered in PROSPERO.

## ELIGIBILITY CRITERIA

### Participants and target condition

Studies evaluating clinical or archived *Mycobacterium tuberculosis* complex isolates from MDR-TB or pre-XDR-TB suspected/enriched populations were included. Both susceptible and resistant isolates reported within these studies were considered for diagnostic accuracy analysis. Both primary and acquired resistance patterns were eligible for consideration. Studies involving non-tuberculosis mycobacteria or lacking sufficient data for this review were excluded.

### Index test

The index test was phenotypic drug susceptibility testing performed exclusively using the BMD method with the Sensititre MYCOTB AST plate because of the methodological heterogeneity among studies and differing interpretative criteria. Studies employing alternative broth microdilution platforms (e.g., MYCOTBI, UKMYC5/UKMYC6, and CRyPTIC plates) or other phenotypic methods as the index test were excluded from the analysis.

### Reference standard

Eligible reference standards included all three WHO-endorsed phenotypic DST methods, including the BACTEC MGIT 960 liquid culture system, the Lowenstein–Jensen proportion method, and the indirect agar proportion method on Middlebrook 7H10 medium.

### Study design, setting, and reporting characteristics

Non-randomized studies were included, comprising prospective and retrospective observational studies, comparative analyses, pilot studies, and cross-sectional diagnostic accuracy studies. Randomized controlled trials, case series, case reports, narrative reviews, and methodological studies without comparative diagnostic data were excluded. No language restrictions were applied. Studies published between January 2010 and December 2025 were considered.

### Information sources and search strategy

Both published and unpublished studies were sought. Indexed databases queried included PubMed, One Nation One Subscription (ONOS), Scopus, ScienceDirect, Web of Science, Cochrane, and Google Scholar. The final search was conducted up to December 2025. Comprehensive search strategies using Boolean operators and MeSH terms were developed for each database. The primary search terms included (“broth microdilution” OR “Sensititre MYCOTB”) AND (“Mycobacterium tuberculosis” OR MDR-TB OR “XDR-TB”) to get the relevant studies. Search strategies were tailored to each database indexing system.

### Study selection

Two phases of study selection were carried out. Titles and abstracts were evaluated separately by two reviewers to identify papers that could qualify by using the Rayyan software. Only studies reporting sufficient diagnostic accuracy data in the form of 2 × 2 contingency tables (TP, FP, FN, TN) were included for pooled estimate calculation in the meta-analysis. Studies limited to methodological descriptions or agreement analysis without extractable diagnostic accuracy data were excluded, even if they evaluated the Sensititre MYCOTB assay. After that, full-text publications were evaluated according to predetermined eligibility criteria. Discrepancies were resolved through discussion, and unresolved issues were referred to a third reviewer when consensus could not be established. The PRISMA 2020 flow diagram is used to display the research selection procedure.

### Data extraction

Data retrieval was performed individually by two reviewers using a predefined and piloted data retrieval form. Extracted data included country, publication year, and author name, sample size, comparison method, drugs included in the study, drugs excluded from the study, and plates used. The corresponding authors were contacted to obtain or clarify missing or unclear data. The target condition was phenotypic resistance in *M. tuberculosis* complex isolates classified as MDR-TB or pre-XDR-TB. Diagnostic outcomes were extracted at the per-isolate level. Two reviewers used the QUADAS-2 methodology to independently evaluate risk of bias (ROB) and application problems in all four domains. Table 1 clarifies how the reference method has been interpreted and provides the critical concentrations and the guidelines used to interpret them. Table 2 describes how the Sensititre MYCOTB plate results have been evaluated compared to the reference method. For the meta-analysis, only the final categorical outcomes from each study were extracted and presented as 2 × 2 contingency tables (TP, FP, FN, TN). Diagnostic accuracy was evaluated using these study-level categorical agreement data.

**TABLE 1:** Critical concentrations (CCs) and reference guidelines used across the included studies evaluating reference standards^*a*^^,*b*,^*^c^*

Study	Guidelines used	INH	RMP	RBT	EMB	OFX	MFX	SM	AMK	KM	CS	ETH	PAS
Hall et al. (13)	CLSI M24-A2 (2011)	0.2, 1.0	1.0	0.5	5.0, 10.0	2.0	2.0	2.0, 10.0	5.0	5.0	25.0	5.0	2.0
Mpagama et al. (14)	Previously published studies	0.1	1.0	NA	NA	NA	NA	1.0	NA	NA	NA	5.0	NA
Lee et al. (4)	CLSI M24-A2 (2011)	0.2, 1.0	1.0	0.5	5.0, 10.0	2.0	0.5, 2.0	2.0, 10.0	4.0	5.0	25.0	5.0	2.0
Heysell et al. (15)	BACTEC MGIT 960 DST protocol (specifically not mentioned); additional APM breakpoint comparison derived from CLSI M24-A2	0.1	1.0	0.5	5.0	2.0	0.25, 0.5	1.0	NA	2.5	NA	5.0	2.0
Nosova et al. (11)	Previously published studies	0.2	1.0	0.5	5.0	2.0	0.5	2.0	4.0	5.0	30	5.0	2.0
Yu et al. (8)	CLSI M24 A2 (2011)	0.2	40	20	2.0	2.0	1.0	4.0	30	30	40	40	1.0
Foongladda et al. (12)	Site-specific standards: Thailand: APM; Bangladesh: LJ proportion; Tanzania: MGIT 960	APM 0.2/LJ 0.2/MGIT 0.1	APM 1/LJ 40/MGIT 1	NA	APM 5/LJ 2/MGIT 5	APM 2/LJ 2/MGIT 2	APM 0.5/MGIT 0.25	APM 2/LJ 4/MGIT 1	APM 4	APM 5/LJ 30/MGIT 2.5	NA	APM 5/LJ 40/MGIT 5	APM 2/MGIT 2
Abdel-Rahman et al. (16)	CLSI M24 A2 (2011)	0.2, 1.0	40	20	2.0	2.0	1.0	4.0	30	30	40	40	1.0
Xia et al. (6)	CLSI M24 A2 (2011)	0.2, 1.0	1.0	0.5	5.0, 10.0	2.0	0.5, 2.0	2.0, 10.0	4.0	5.0	25.0	5.0	2.0
Varıcı Balcı et al. (7)	CLSI M24 A2 (2011)	0.2, 1.0	1.0	0.5	5.0, 10.0	2.0	0.5, 2.0	2.0, 10.0	4.0	5.0	25.0	5.0	2.0
Ssengooba et al. (5)	BACTEC MGIT 960 DST protocol; additional APM breakpoint comparison derived from CLSI M24-A2	0.1	1.0	NA	5.0	NA	NA	1.0	NA	NA	NA	NA	NA
Martin et al. (17)	Previously published studies	0.1	1.0	NA	5.0	NA	NA	NA	NA	NA	NA	NA	NA
Jaglal et al. (18)	CLSI M24-A2 (2011)	0.2	1.0	NA	NA	2.0	2.0	NA	NA	5.0	NA	NA	NA
Ceyhan et al. (19)	CLSI M24 A2 (2011)	0.2	40	20	2.0	2.0	1.0	4.0	30	30	40	40	1.0

^*a*^ All the critical concentration (CC) values mentioned in the unit of µg/mL.

^*b*^ In the Hall et al. (13) study, amikacin CC is reported as 5.0 µg/mL, which differs from the CLSI M24; the reason is not clearly stated.

^*c*^ NA = Not available.

**TABLE 2:** Methodological approaches used by included studies for interpreting Sensititre MYCOTB MIC results against phenotypic reference standards^*a*^^,*b*^

Study	Reference standard used	How MYCOTB-susceptible isolates were defined	How MYCOTB-resistant isolates were defined	Handling of non-matching CCs/breakpoints	How PAS and CS CCs were derived despite the absence of WHO-endorsed CCs
Hall et al. (13)	1% indirect agar proportion method (APM) on Middlebrook 7H10 medium	MYCOTB MIC equivalent to or lower than the APM critical concentration	MYCOTB MIC is higher than the APM critical concentration.	Nearest MYCOTB concentration within ±1 doubling dilution was considered equivalent for categorical interpretation. For CS, APM CC 25 µg/mL corresponded to MYCOTB 32 µg/mL. PAS used the same concentration (2 µg/mL) in both methods.	PAS and CS CCs were derived from historical APM/CLSI laboratory reference standards rather than WHO-endorsed CC guidelines.
Mpagama et al. (14)	BACTEC MGIT phenotypic DST	MYCOTB MIC at or below the predefined resistance breakpoint.	MYCOTB MIC above the predefined resistance breakpoint.	Resistance breakpoints were adopted from earlier MYCOTB validation studies and Middlebrook 7H9 liquid media CCs. For drugs without exact WHO-endorsed CCs, operational MYCOTB MIC thresholds were used directly.	PAS breakpoint was defined as >2.0 µg/mL and CS breakpoint as >32 µg/mL using previously published MYCOTB/APM laboratory standards rather than WHO-endorsed CCs.
Lee et al. (4)	Middlebrook 7H10 agar proportion method (APM)	MYCOTB MIC at or below the APM critical concentration (CC).	MYCOTB MIC above the APM critical concentration (CC).	“Conditional agreement” of ±1 doubling dilution around the APM CC was used for drugs where MYCOTB concentrations did not exactly correspond to APM CCs (e.g., EMB, SM, CS). PAS and CS were interpreted using historical APM CCs (PAS: 2 µg/mL; CS: 25 µg/mL).	PAS and CS CCs were based on historically accepted CLSI/APM laboratory critical concentrations used in conventional phenotypic DST laboratories before subsequent WHO standardization updates.
Heysell et al. (15)	BACTEC MGIT 960 phenotypic DST comparator	MYCOTB MIC at or below the selected breakpoint concentration	MYCOTB MIC above selected breakpoint concentration	When MGIT 960 and APM breakpoints differed, both breakpoints were analyzed separately. Borderline interpretation zones of ±1 dilution around the APM breakpoint were also evaluated.	PAS and CS breakpoints were operational MIC thresholds derived from prior APM/MYCOTB validation literature rather than WHO-endorsed CC tables.
Nosova et al. (11)	BACTEC MGIT 960 phenotypic DST comparator	MYCOTB MIC at or below the MGIT 960 critical concentration (CC).	MYCOTB MIC above the MGIT 960 critical concentration (CC).	MYCOTB resistance interpretation used predefined MYCOTB breakpoints: STR 2.0, RMP 1.0, RFB 0.5, INH 0.2, EMB 5.0, OFX 2.0, MFX 0.5, KAN > 5.0, AMK > 4.0, PAS 2, ETH 5, and CS 30 µg/mL.	PAS and CS breakpoints were adopted from previously established MYCOTB/APM literature rather than the WHO-endorsed CC guidelines.
Yu et al. (8)	Lowenstein–Jensen (LJ) proportion method	MYCOTB MIC at or below the LJ critical concentration (CC).	MYCOTB MIC above the LJ critical concentration (CC).	MYCOTB resistance was interpreted using predefined MIC cut-offs. For drugs lacking standardized CCs, locally validated operational breakpoints were applied.	PAS and CS CCs were derived from historical LJ proportion method practices and previously published MYCOTB validation studies.
Foongladda et al. (12)	Agar proportion method (APM), Lowenstein–Jensen (LJ) proportion method, and BACTEC MGIT 960 phenotypic DST.	MYCOTB MIC at or below the method-specific critical concentration (CC).	MYCOTB MIC above the method-specific critical concentration (CC).	The study did not apply a single universal breakpoint system. Instead, method-specific CCs from APM, LJ, or MGIT were applied according to the participating laboratory. The agar proportion CC was generally used as the MYCOTB breakpoint for interpretation. After local categorical interpretation, all results were pooled into combined 2 × 2 analyses. Therefore, pooled analyses reflected categorical agreement rather than direct MIC equivalence across standardized breakpoints.	PAS and CS interpretations were based on operational MYCOTB MIC thresholds and local phenotypic DST standards rather than universally accepted WHO standards.
Abdel-Rahman et al. (16)	Lowenstein–Jensen (LJ) proportion method	MMYCOTB MIC at or below the LJ critical concentration (CC).	MYCOTB MIC above the LJ critical concentration (CC).	MYCOTB MIC values across dilution ranges were directly compared with APM categories. No recalculated breakpoint strategy was proposed for drugs lacking exact WHO CCs.	PAS and CS interpretations were based on manufacturer-defined MYCOTB concentration ranges and conventional APM laboratory standards rather than WHO-endorsed CCs. Cycloserine showed the lowest categorical agreement.
Xia et al. (6)	Middlebrook 7H10 agar proportion method (APM)	MYCOTB MIC at or below the APM critical concentration (CC).	MYCOTB MIC above the APM critical concentration (CC).	“Conditional agreement” was applied when MYCOTB MIC was within ±1 doubling dilution of the APM CC. This was used to manage discrepancies between APM and MYCOTB.	PAS (2.0 µg/mL) and CS (25 µg/mL) CCs were derived from CLSI/APM-established laboratory breakpoints used in conventional phenotypic DST rather than WHO-endorsed CCs. Sequencing for discrepant PAS/CS isolates was not performed because resistance mechanisms for these drugs were unclear.
Varici Balci et al. (7)	Middlebrook 7H10 agar proportion method (APM)	MYCOTB MIC at or below the APM critical concentration (CC).	MYCOTB MIC above the APM critical concentration (CC).	The MYCOTB plate contained predefined dilution ranges for PAS and CS, and interpretation was directly compared with CLSI-based APM categories. No alternative breakpoint recalibration was proposed.	PAS and CS critical concentrations were adopted from CLSI/APM laboratory standards and manufacturer-defined MYCOTB concentration ranges rather than WHO-endorsed CCs.
Ssengooba et al. (5)	BACTEC MGIT 960 phenotypic DST protocol with additional agar proportion method (APM) breakpoint comparison	MYCOTB MIC at or below the MGIT 960 critical concentration (CC).	MYCOTB MIC above the MGIT 960 critical concentration (CC).	For drugs with two concentrations, only the higher concentration was used for agreement analysis. MYCOTB breakpoints were directly compared against LJ and MB7H10 reference methods.	PAS and CS were not evaluated because only first-line drugs (STR, INH, RIF, EMB) were included in the study.
Martin et al. (17)	BACTEC MGIT 960 phenotypic DST	MYCOTB MIC at or below the MGIT 960 critical concentration (CC)	MYCOTB MIC above the MGIT 960 critical concentration (CC).	The study specifically addressed drugs whose exact CCs were absent on MYCOTB plates (e.g., INH, EMB). The nearest available MYCOTB dilution and MIC interpretation around CC values were used for categorical comparison.	PAS and CS were not evaluated in this study because only EMB, INH, and RIF were analyzed. Therefore, no PAS/CS breakpoint derivation was performed.
Jaglal et al. (18)	Middlebrook 7H10 agar proportion method (APM)	MYCOTB MIC at or below the APM critical concentration (CC).	MYCOTB MIC above the APM critical concentration (CC).	The study mainly evaluated categorical agreement and MIC distributions. MYCOTB dilution ranges were interpreted in relation to APM susceptibility categories.	PAS and CS were included on MYCOTB plates, but formal WHO-endorsed CC derivation was not discussed. Interpretations relied on standard MYCOTB manufacturer-defined MIC ranges and conventional laboratory DST practices.
Ceyhan et al. (19)	Lowenstein–Jensen (LJ) proportion method	MYCOTB MIC at or below the LJ critical concentration (CC).	MYCOTB MIC above the LJ critical concentration (CC).	Exact final drug concentrations for LJ method were predefined and directly compared with MYCOTB MIC distributions. No alternative recalibration strategy was proposed.	PAS and CS final LJPM concentrations were explicitly defined as 1.0 µg/mL and 40.0 µg/mL, respectively, using laboratory LJ proportion standards rather than WHO-endorsed CC updates.

^*a*^ Historical CLSI/APM critical concentrations refer to operational drug concentrations previously used in conventional agar proportion DST protocols and CLSI M24 guidelines before later WHO standardization updates.

^*b*^ In the study by Foongladda et al. (12), pooled 2 × 2 analyses represented categorical agreement across different reference methods (LJ, MGIT, and APM), each using method-specific critical concentrations rather than standardized MIC breakpoints.

### Diagnostic accuracy measures and data synthesis

The primary diagnostic accuracy measures were sensitivity and specificity, derived from 2 × 2 contingency tables. Secondary measures included likelihood-based diagnostic measures, diagnostic OR, forest plot, and the area under the hierarchical summary receiver operating characteristic (HSROC) curve. Meta-analysis was executed using MetaBayes-DTA and Review Manager (RevMan) version 5.4. Aggregated estimates were generated using bivariate mixed-effects or Bayesian HSROC models. HSROC curves were constructed for each drug. Sensitivity analysis was additionally performed for moxifloxacin, para-aminosalicylic acid, and cycloserine because they produce low sensitivity values. Studies containing fewer than two resistant isolates in the reference standard were excluded to evaluate the influence of extremely small resistant sample sizes on pooled diagnostic accuracy estimates and heterogeneity.

### Certainty of evidence

The GRADE method for diagnostic accuracy studies was used to evaluate the degree of confidence of evidence for each diagnostic result, supported by the GRADEpro DTA tool and summarized in a summary of findings table.

### Study characteristics

**Fig 1:**
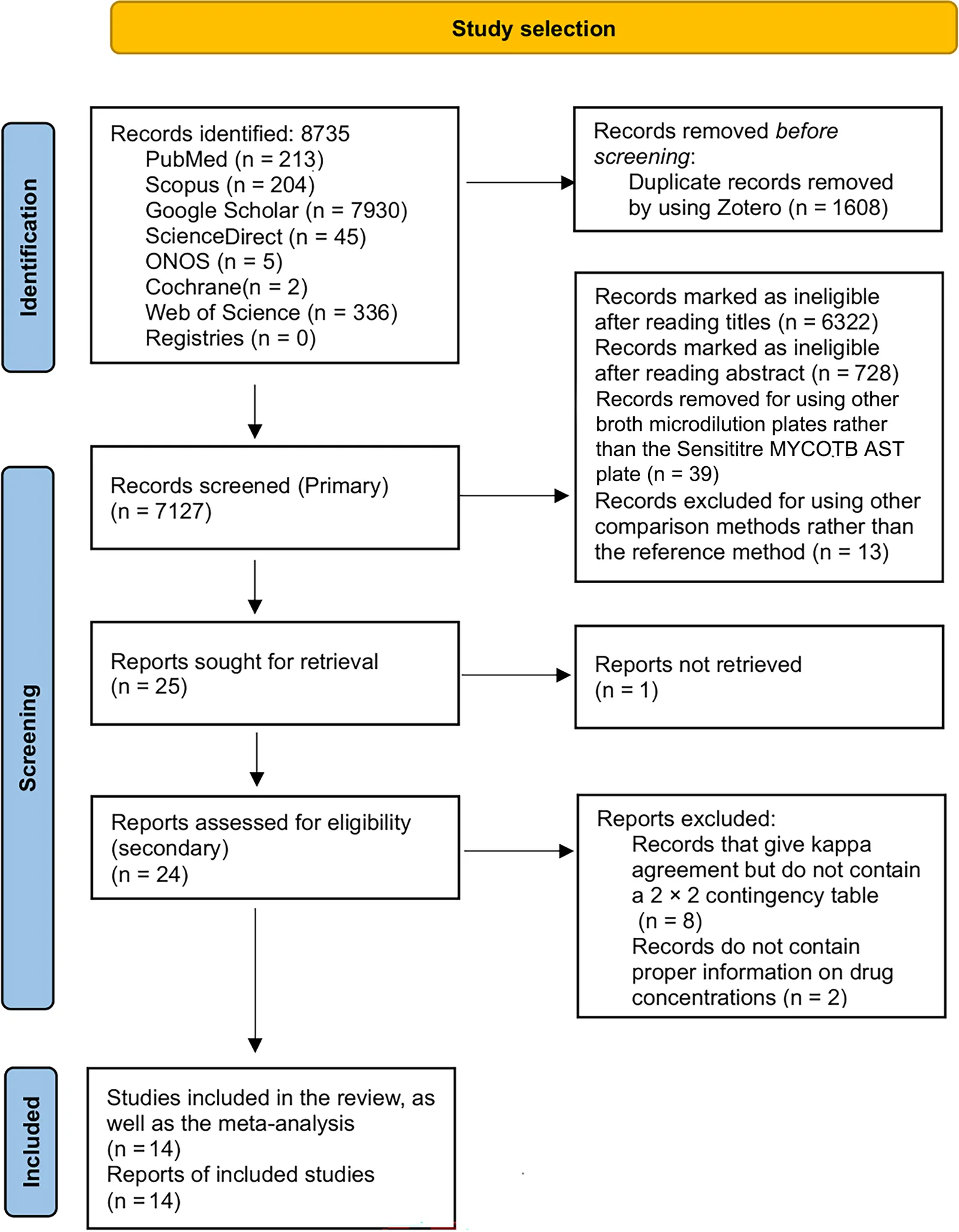
Literature selection flowchart. Note: Meta-analysis requires TP, TN, FP, and FN data to generate forest plots and HSROC curves in diagnostic accuracy studies. Therefore, the articles that do not provide a 2 × 2 table have been excluded, even if they are related to our title. Zotero software is used to identify duplicate studies by importing RIS files. Two reviewers independently performed abstract screening and full-text assessment using Rayyan software. A third reviewer resolved disagreements between the reviewers.

The two writers appraised every article and included it in the review for the reasons listed in the PRISMA flowchart below (Fig. 1). All enrolled studies contribute to determining the diagnostic accuracy of Sensititre MYCOTB relative to the reference method for first- and second-line drugs by constructing 2 × 2 contingency tables, constructing forest plots, and performing HSROC analyses, thereby advancing the review objectives. The basic information about the studies, including the geographic origin of the samples and the comparative methods for each study, is presented in Table 3.

**TABLE 3:** Descriptive characteristics of included studies*^a^*^,^*^b^*^,^*^c^*

S. no.	Studies included	Country	Sample size	Comparison method	Drugs included in study	Drugs excluded In study	Plate used in the study
1	Hall et al. (13)	USA	122 (MTBC)	Agar proportion method MB7H10	INH, RMP, RBT, EMB, OFX, MFX, SM, AMK, KM, CS, ETH, PAS	–	Original MYCOTB plate
2	Mpagama et al. (14)	Tanzania	22 (MTBC)	BACTEC MGIT 960	RMP, INH, EMB, SM	RBT, OFX, MFX, AMK, KM, CS, ETH, PAS	Original MYCOTB plate
3	Lee et al. (4)	New Jersey, USA	222 (MTBC)	Agar proportion method MB7H10	INH, RMP, RBT, EMB, OFX, MFX, SM, AMK, KM, CS, ETH, PAS	–	Original MYCOTB plate
4	Heysell et al. (15)	Moshi, Tanzania	141 (MTBC)	BACTEC MGIT 960	INH, RMP, RBT, EMB, OFX, MFX, SM, KM, ETH, PAS	AMK, CS	Original MYCOTB plate
5	Nosova et al. (11)	Moscow, Russia	144 (MTBC)	BACTEC MGIT 960	INH, RMP, EMB, OFX, MFX, SM, AMK, KM, ETH, PAS	RBT, CS	Original MYCOTB plate
6	Yu et al. (8)	Beijing, China	126 (MTBC)	LJ proportion method	INH, RMP, RBT, EMB, OFX, MFX, SM, AMK, KM, CS, ETH, PAS	–	Original MYCOTB plate
7	Foongladda et al. (12)	Bangladesh, Thailand, and Tanzania	212 (MTBC)	Agar proportion method, MB7H10, LJ proportion method, BACTEC MGIT 960	INH, RMP, EMB, OFX, MFX, SM, AMK, KM, ETH, PAS	CS, RBT	Original MYCOTB plate
8	Abdel Rahman et al. (16)	Egypt	40 (MTBC)	LJ proportion method	INH, RMP, RBT, EMB, OFX, MFX, SM, AMK, KM, CS, ETH, PAS	–	Original MYCOTB plate
9	Xia et al. (6)	Beijing, China	193 (MTBC)	Agar proportion method MB7H10	INH, RMP, RBT, EMB, OFX, MFX, SM, AMK, KM, CS, ETH, PAS	–	Original MYCOTB plate
10	Varici Balci et al. (7)	Izmir, Turkey	100 (MTBC)	Agar proportion method MB7H10	INH, RMP, RBT, EMB, OFX, MFX, SM, AMK, KM, CS, ETH, PAS	–	Original MYCOTB plate
11	Ssengooba et al. (5)	Kampala, Uganda	76 (MTBC)	Agar proportion method MB7H10, and the LJ proportion method	INH, RMP, EMB, SM	RBT, OFX, MFX, AMK, KM, CS, ETH, PAS.	Original MYCOTB plate
12	Martin et al. (17)	USA	142 (MTBC)	BACTEC MGIT 960	RMP, INH, EMB	RBT, OFX, MFX, SM, AMK, KM, CS, ETH, PAS	Original MYCOTB plate
13	Jaglal et al. (18)	Durban, South Africa	134 (MTBC)	Agar proportion method MB7H10	INH, RMP, OFX, MFX, KM	RBT, EMB, SM, AMK, CS, ETH, PAS	Original MYCOTB plate
14	Ceyhan et al. (19)	Turkey	100 (MTBC)	LJ proportion method	INH, RMP, RBT, EMB, OFX, MFX, SM, AMK, KM, CS, ETH, PAS	–	Original MYCOTB plate

^*a*^ LJ, Lowenstein–Jensen; MB, Middlebrook; INH, isoniazid; RMP, rifampicin; RBT, rifabutin; EMB, ethambutol; OFX, ofloxacin; MFX, moxifloxacin; SM, streptomycin; AMK, amikacin; KM, kanamycin; CS, cycloserine; ETH, ethionamide; PAS, para-aminosalicylic acid; MTBC, *Mycobacterium tuberculosis* complex; MTB, *Mycobacterium tuberculosis*.

^*b*^ In Ssengooba et al.'s 2018 study (5), they compared both the LJ proportion method and the agar proportion Middlebrook 7H10 method. Still, we included only the agar proportion MB7H10 method data in this review because it provides faster growth and earlier detection of MTBC than the LJ proportion method. If we include both results in the analysis, it will increase the duplicate sample size.

^*c*^ In some studies, the authors used different sample sizes for different drugs, for example, Jaglal et al.'s study (18).

### Bias assessment and applicable problems

**Fig 2:**
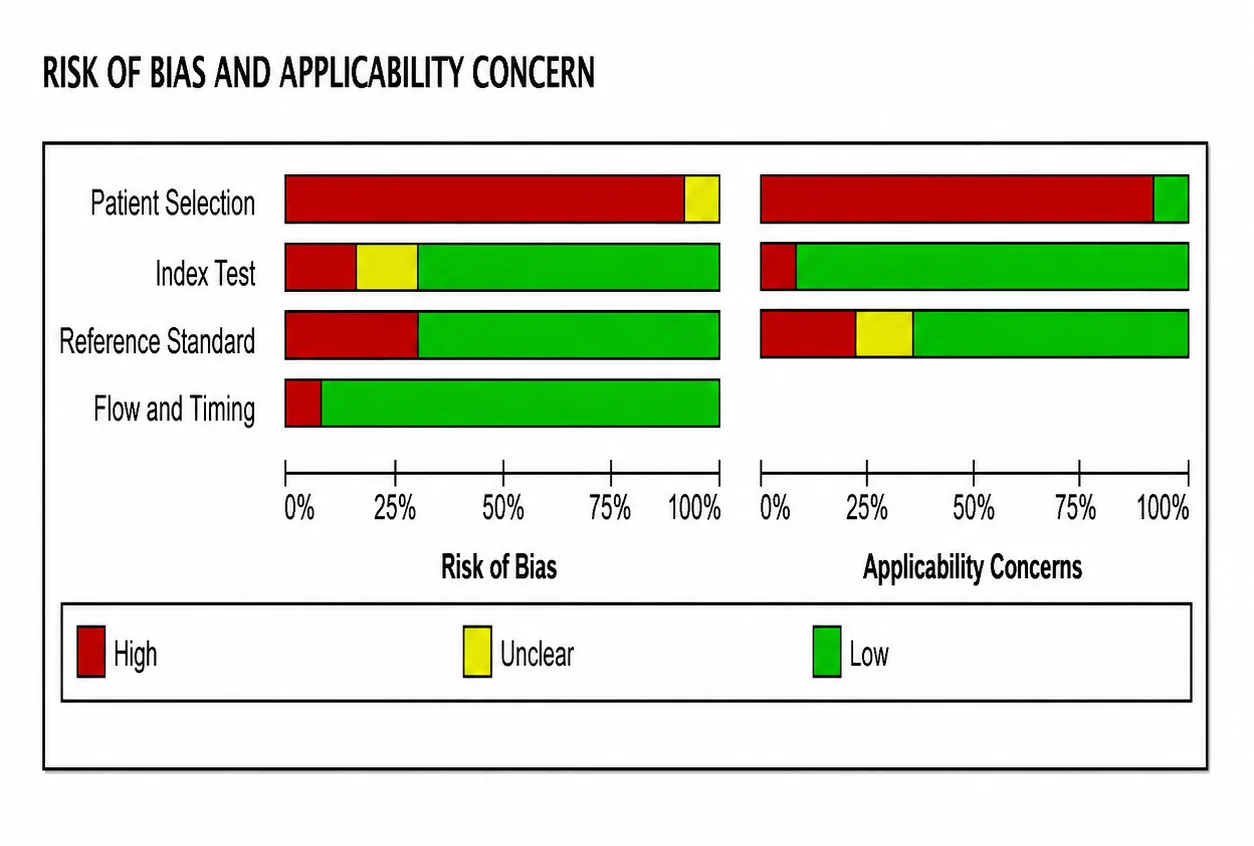
Bias assessment and application problem. Note: The risk of bias and applicability concerns were generated by REVMAN 5.4. Most studies showed a high risk of bias in patient selection due to the use of pre-selected or drug-resistant isolates.

Figure 2 interpretation primarily begins with the selection of high-risk patients in each study due to non-consecutive sampling and the use of archived isolates. This may affect heterogeneity across studies of all the drugs. In other domains, such as index test, flow and timing, and the reference standard, along with the entire study information, there is an insignificant risk of bias, with only a limited number of studies showing unclear risk of bias. This bias assessment contributes significantly to the internal validity of this review. However, applicability concerns focus on external validity, and the fact that most studies showed minimal risk of bias suggests that the studies included in the review are relevant to the context. Table 4 describes the concentrations across different reference methods from the WHO protocols (20–22).

**TABLE 4:** Critical concentration (CCs) and clinical breakpoints (CBs) for antitubercular drugs across Lowenstein–Jensen (LJ), Middlebrook 7H10, and BACTEC MGIT 960 systems according to the WHO 2018 and 2021 guidelines (20–22)*^a^*^,^*^b^*^,^*^c^*

Drug	2018 LJ CC	2018 7H10 CC	2018 MGIT CC	2018 CB	2021 LJ CC	2021 7H10 CC	2021 MGIT CC	2021 CB
INH	0.2	0.2	0.1	NA	0.2	0.2	0.1	NA
RMP	40	1.0	1.0	NA	40	0.5	0.5	NA
RBT	NA	NA	NA	NA	NA	0.5	0.5	1.0
EMB	2.0	5.0	5.0	NA	NA	NA	NA	NA
OFX	2.0	2.0	2.0	NA	NA	NA	NA	NA
MFX (CC)	1.0	0.5	0.25	NA	NA	NA	NA	NA
MFX (CB)	2.0	2.0	1.0	Available	NA	NA	NA	Available
SM	4.0	2.0	1.0	NA	NA	NA	NA	NA
AMK	30	2.0	1.0	NA	NA	NA	NA	NA
KM	30	5.0	2.5	NA	NA	NA	NA	NA
CS	NA	NA	NA	NA	NA	NA	NA	NA
ETH	40	5.0	5.0	NA	NA	NA	NA	NA
PAS	NA	NA	NA	NA	NA	NA	NA	NA

^*a*^ LJ, Lowenstein–Jensen; MB, Middlebrook; MGIT 960, Mycobacterium growth indicator tube 960; INH, isoniazid; RMP, rifampicin; RBT, rifabutin; EMB, ethambutol; OFX, ofloxacin; MFX, moxifloxacin; SM, streptomycin; AMK, amikacin; KM, kanamycin; CS, cycloserine; ETH, ethionamide; PAS, para-aminosalicylic acid; CC, critical concentrations; CB, clinical breakpoints; NA, not available.

^*b*^ WHO 2021 guidelines only contain the information about rifampicin, isoniazid, and rifabutin (22).

^*c*^ Table 4 was included for comparative reference only and to demonstrate the evolution of critical concentrations across methods. However, the meta-analysis did not apply the unified WHO breakpoints, as the included studies used their own contemporaneous CLSI-, WHO-, or study-specific interpretative criteria, and only the published categorical 2 × 2 data were pooled.

### Hierarchical summary receiver operating characteristics curve analysis

**Fig 3:**
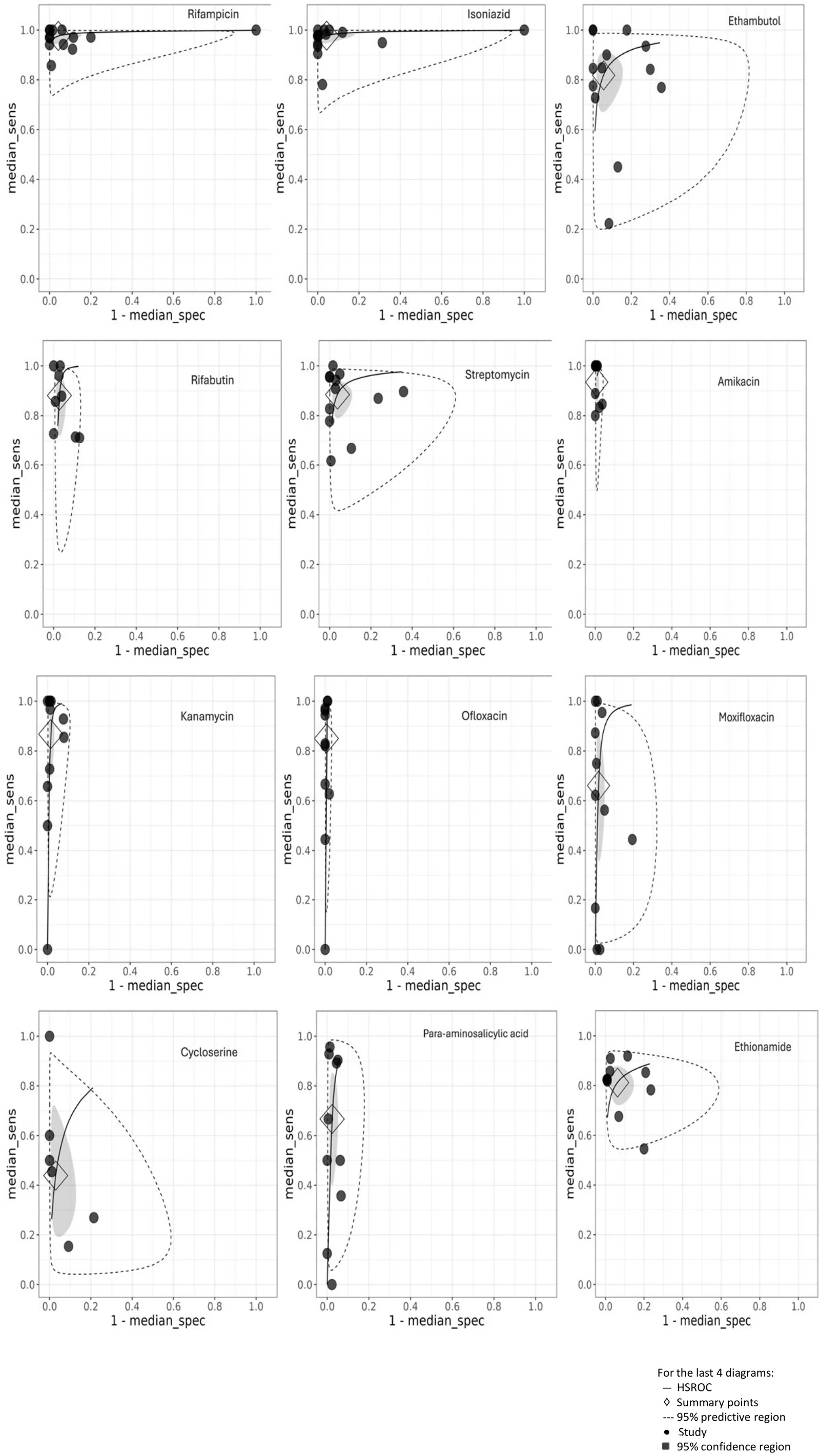
HSROC plots of hierarchical Bayesian median sensitivity and specificity estimates. Note: In some studies, the absence of TP or TN values precluded the calculation of sensitivity and specificity; therefore, these studies were excluded from the HSROC analysis. All the plots were designed by MetaBayes-DTA. In the HSROC plots, the axes represent hierarchical Bayesian median sensitivity and median specificity estimates automatically generated by the MetaBayesDTA model from the posterior distributions of the included studies.

**Fig 4:**
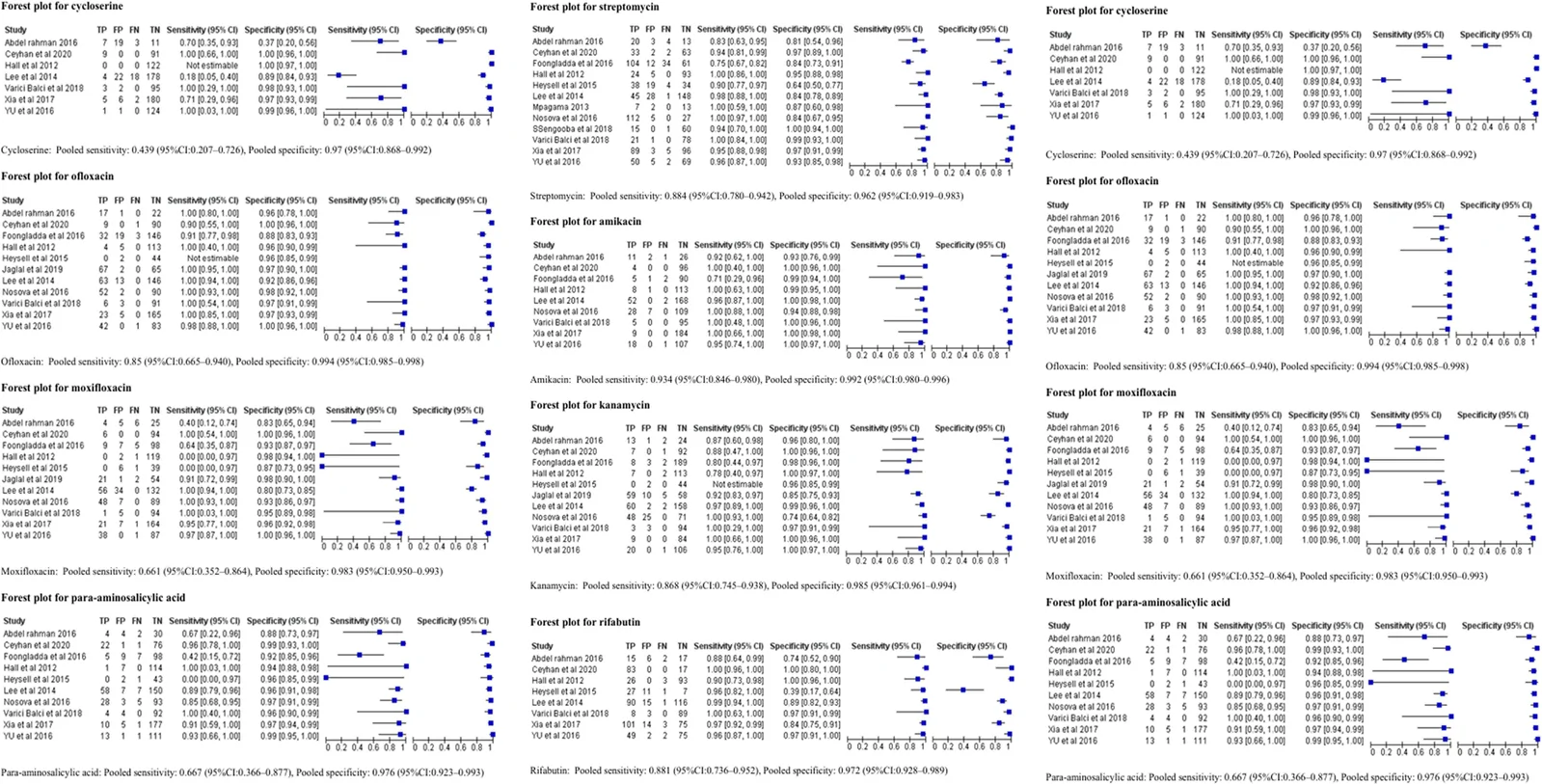
Forest plots of sensitivity and specificity for 12 anti-tubercular drugs.

Quantitative values of pooled estimates validate the entire analysis in Fig. 3 and 4. The HSROC plots were generated using the MetaBayesDTA hierarchical Bayesian model. Each circle represents an individual study estimate within the hierarchical Bayesian model. The HSROC curves of the first-line drugs, especially rifampicin and isoniazid, are very close to the top-left corner, with narrow confidence and prediction regions, indicating higher diagnostic accuracy. For the drug ethambutol, its curve steepens downward, and it has a wider predictive region because of different thresholds used across studies. Still, overall diagnostic performance is acceptable, given its excellent sensitivity, specificity, and diagnostic odds ratio. Rifabutin demonstrated results comparable to rifampicin, but with greater variability among the included studies.

The resistance profile of the second-line drugs is the most critical thing to determine pre-XDR-TB. Injectable drugs, such as amikacin and kanamycin, have their curves in the left top quadrant, similar to rifampicin. However, this is not the case for the predictive region, which is wider but still exhibits good diagnostic accuracy, as supported by its statistical values. Meanwhile, streptomycin and ethionamide share characteristics with ethambutol and have moderate diagnostic accuracy. Fluoroquinolones demonstrate good diagnostic accuracy performance as supported by their curves, except for the notable heterogeneity of moxifloxacin, which may compromise the sensitivity of finding resistance for moxifloxacin. Cycloserine and para-aminosalicylic acid exhibit poor diagnostic performance due to high heterogeneity and wide threshold variability across studies.

### Significance of pooled estimates and forest plots

Forest plots (Fig. 4) summarize the sensitivity and specificity estimates for each antimicrobial across the included studies. From Table 5, the aggregated estimates from 14 studies (*n* = 1,728) displayed that rifampicin and isoniazid have high-quality sensitivities (0.976 [95% CI: 0.94–0.99] and 0.977 [95% CI: 0.95–0.99]) and specificities (0.958 [95% CI: 0.84–0.98] and 0.957 [95% CI: 0.83–0.99]) among all drugs, confirming good discriminatory power. In contrast, cycloserine, moxifloxacin, and para-aminosalicylic acid exhibited comparatively lower sensitivities (0.439 [95% CI: 0.20–0.72], 0.661 [95% CI: 0.35–0.86], and 0.667 [95% CI: 0.40–0.83], respectively), all of which were lower than those of all the drugs, demonstrating a lower ability to detect resistant samples. Among second-line agents, amikacin showed the highest performance, with a sensitivity of 0.934 (95% CI: 0.84–0.98) and specificity of 0.992 (95% CI: 0.98–0.99), followed by kanamycin and ofloxacin. Rifabutin showed similar sensitivity to ethambutol 0.88 [95% CI: 0.73–0.95] and to streptomycin, while ethionamide exhibited moderate and acceptable diagnostic accuracy. Regarding heterogeneity, rifampicin, isoniazid, rifabutin, and amikacin have their study points clustered, indicating low heterogeneity and increasing confidence in the pooled results. Ethambutol, ethionamide, streptomycin, cycloserine, para-aminosalicylic acid, and moxifloxacin have their study points more dispersed due to different thresholds and methodological differences, which highlight moderate heterogeneity; they work well in some settings but not in others.

**TABLE 5:** Aggregated diagnostic accuracy of the index test^*a*^

S. no.	Drugs	No. of studies	No. of samples	Overall Sensitivity (95% CI)	Overall specificity (95% CI)	Diagnostic odds ratio (95% CI)	Positive likelihood ratio (+LR) (95% CI)	Negative likelihood ratio (−LR) (95%CI)
1	RIFAMPICIN	14	1,728	0.976 (0.949–0.989)	0.958 (0.904–0.983)	933.577	23.28	0.025
2	ISONIAZID	14	1,728	0.977 (0.951–0.991)	0.957 (0.835–0.990)	944.885	22.798	0.024
3	ETHAMBUTOL	13	1,593	0.818 (0.686–0.907)	0.944 (0.846–0.979)	77.532	14.564	0.194
4	STREPTOMYCIN	12	1,451	0.884 (0.780–0.942)	0.962 (0.919–0.983)	195.296	22.965	0.121
5	AMIKACIN	09	1,145	0.934 (0.846–0.980)	0.992 (0.980–0.996)	1,772.162	117.486	0.066
6	KANAMYCIN	11	1,328	0.868 (0.745–0.938)	0.985 (0.961–0.994)	425.825	55.888	0.134
7	RIFABUTIN	08	951	0.881 (0.736–0.952)	0.972 (0.928–0.989)	259.946	31.23	0.123
8	CYCLOSERINE	07	903	0.439 (0.207–0.726)	0.97 (0.868–0.992)	26.021	14.464	0.583
9	OFLOXACIN	11	1,427	0.85 (0.665–0.940)	0.994 (0.985–0.998)	911.43	133.33	0.151
10	MOXIFLOXACIN	11	1,290	0.661 (0.352–0.864)	0.983 (0.950–0.993)	112.848	37.564	0.346
11	ETHIONAMIDE	10	1,280	0.812 (0.721–0.876)	0.937 (0.858–0.975)	65.433	12.93	0.202
12	PARA-AMINOSALICYLIC ACID	10	1,197	0.667 (0.366–0.877)	0.976 (0.923–0.993)	81.437	27.21	0.342
13	MOXIFLOXACIN (SA)	08	1,022	0.801 (0.585–0.924)	0.976 (0.925–0.992)	166.824	33.344	0.205
14	PARA-AMINOSALICYLIC ACID (SA)	08	1,029	0.76 (0.518–0.894)	0.971 (0.939–0.986)	102.029	25.398	0.248
15	CYCLOSERINE (SA)	05	780	0.436 (0.190–0.725)	0.96 (0.832–0.988)	18.515	10.636	10.636

^*a*^ All data were calculated using MetaBayes-DTA; SA, sensitivity analysis.

### Result of sensitivity analysis

**Fig 5:**
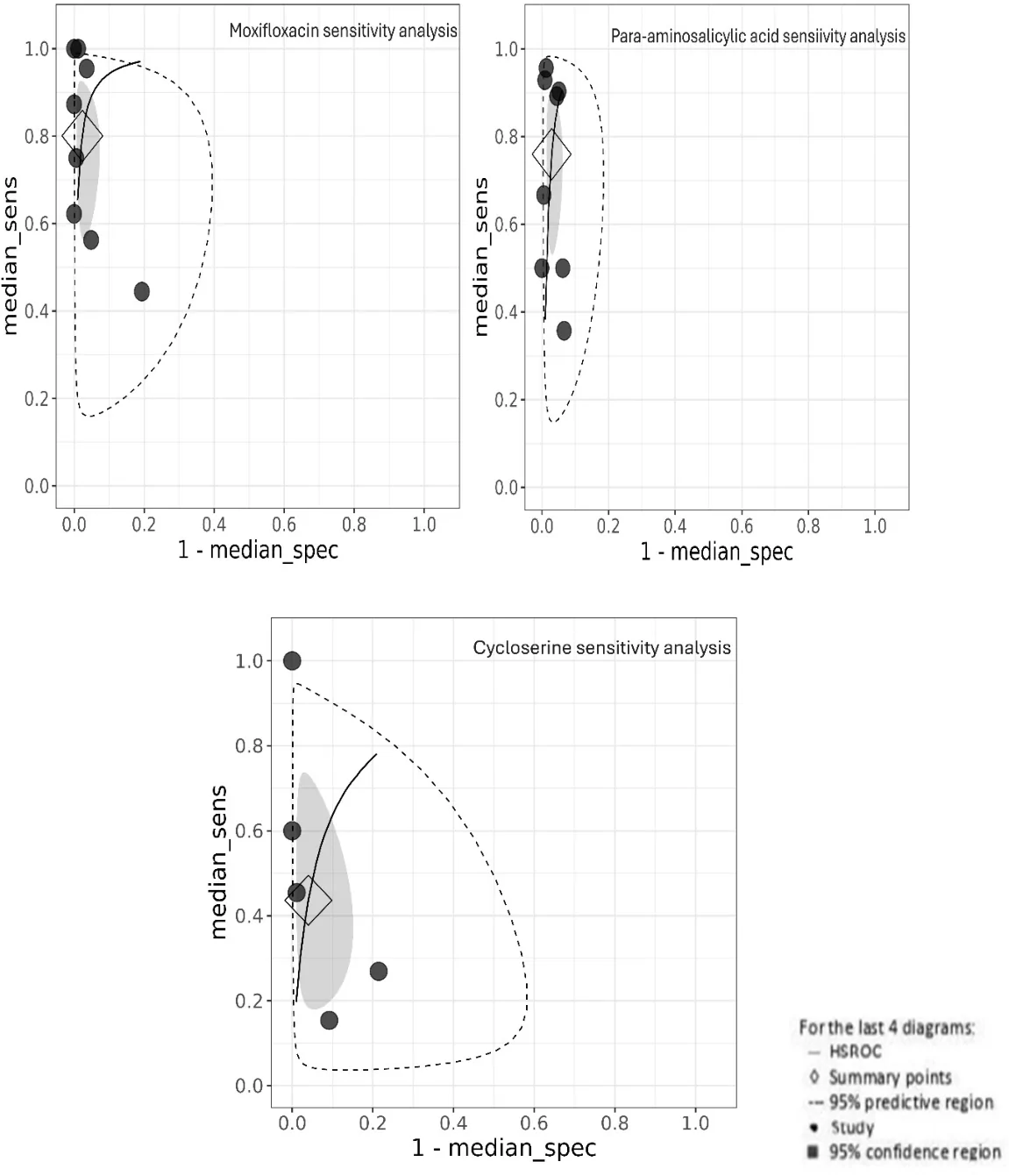
HSROC Bayesian sensitivity analysis of moxifloxacin, para-aminosalicylic acid, and cycloserine. Note: All the circles represent each study. The resistant isolates that are less than two have been removed (*n* = 0, 1). Studies removed for the specific drugs are: moxifloxacin, Hall et al. (13), Varici Balci et al. (7), Heysell et al. (15); para-aminosalicylic acid, Hall et al. (13), Heysell et al. (15); cycloserine, Hall et al. (13), Yu et al. (8). Sensitivity analysis was performed only for these drugs due to their low sensitivity.

Compared with the primary HSROC analysis (Fig. 3), the sensitivity analysis (Fig. 5) demonstrated improved clustering of study estimates and a partial reduction in heterogeneity for moxifloxacin and para-aminosalicylic acid after excluding studies with fewer than two resistant isolates, whereas cycloserine remained unchanged. In the primary analysis, pooled sensitivities for moxifloxacin, para-aminosalicylic acid, and cycloserine were low. Following the sensitivity analysis reported in Table 5, the pooled sensitivity estimates increased to 0.801 (95% CI: 0.585–0.924) for moxifloxacin and 0.76 (95% CI: 0.518–0.894) for para-aminosalicylic acid, whereas cycloserine remained at 0.436 (95% CI: 0.190–0.725), suggesting that factors beyond small resistant sample sizes may contribute to the persistent heterogeneity for those three drugs and low sensitivity observed for cycloserine.

### Certainty of assessment

As reported by GRADEpro GDT, the certainty of assessment results is high for rifampicin, isoniazid, rifabutin, amikacin, and ofloxacin. It suggests that Sensititre MYCOTB may be reliable for drug susceptibility testing. In the other cases, cycloserine and para-aminosalicylic acid indicate low test accuracy, which raises concerns about analytical robustness. For the other drugs, it postulates the moderate certainty of evidence, which is satisfactory and dependable.

### Overall findings

In conclusion, the HSROC analyses and forest plots demonstrate that, with relatively little variability, the Sensititre MYCOTB approach exhibits high, consistent diagnostic accuracy for rifampicin and isoniazid. Other medications, such as amikacin, kanamycin, ofloxacin, rifabutin, streptomycin, ethionamide, and ethambutol, have more varied but acceptable diagnostic accuracy. Although sensitivity analysis demonstrated partial reduction in heterogeneity and improved pooled sensitivity estimates for moxifloxacin and para-aminosalicylic acid, cycloserine continued to show variable performance. Overall, significant heterogeneity and reduced consistency for para-aminosalicylic acid, moxifloxacin, and cycloserine indicate that results for these drugs should be interpreted cautiously and may require confirmation using alternative methods.

### Clinical implications and interpretations

This review and meta-analysis are, to the best of our knowledge, the first to synthesize the diagnostic accuracy of the Sensititre MYCOTB DST for a broad panel of first- and second-line drugs in MDR and pre-XDR TB. Sensititre MYCOTB provides quantitative MICs, which are increasingly recognized as essential for linking phenotypic resistance to pharmacokinetic/pharmacodynamic (PK/PD) targets and for refining clinical breakpoints for *M. tuberculosis* (23–26).

Across 1,700 isolates, Sensititre MYCOTB showed high sensitivity and specificity for rifampicin and isoniazid and good performance for second-line injectables, supporting its use in designing MDR and pre-XDR regimens and individualizing dosing when MICs approach PK/PD thresholds. EUCAST now endorses MIC-based methods, with WHO as the reference for defining epidemiological cut-offs (ECOFFs) and calibrating routine DST methods, and BMD plates, such as MYCOTB or UKMYC6, are central to this effort (9, 23, 25).

One of the main reasons for clinical differences is phenotype-genotype discordance. Certain mutations (such as low-level INH or EMB variants) yield MICs near critical concentrations, according to studies combining BMD MICs with whole-genome sequencing (WGS). This results in inconsistent “susceptible/resistant” calls across techniques and breakpoints (25, 27, 28). BMD helps resolve such “borderline” resistance and supports more nuanced decisions (e.g., dose escalation vs drug replacement) (25, 27, 29). Discordance between phenotypic and genotypic DST is also driven by phenotypically missed but genotypically detected (27, 30, 31). These phenomena are common in MDR/pre-XDR cohorts and are associated with discrepancies and worse outcomes (27, 30, 31).

Our findings that cycloserine, moxifloxacin, and para-aminosalicylic acid show high specificity but inconsistent sensitivity likely reflect both intrinsic methodological variability and uncertainty in the relationship between laboratory MIC values and clinically achievable drug concentrations, together with breakpoint-related issues, similar to problems described for ethambutol, ethionamide, and some fluoroquinolones (23, 28, 29, 32, 33). These inconsistencies partly arise because several drugs lack universally harmonized MIC breakpoints across LJ, MGIT, and agar proportion reference methods, and some included studies relied on historical CLSI/APM operational critical concentrations or locally validated thresholds rather than WHO-standardized MIC breakpoints, which were not available at the time the studies were conducted. Although the sensitivity analysis demonstrated improved pooled sensitivity estimates for moxifloxacin and para-aminosalicylic acid after excluding studies with very small numbers of resistant isolates, moderate heterogeneity, wide confidence intervals, and a limited number of available studies for cycloserine, the issues that persisted across studies were not fully resolved. Compared with rifampicin, isoniazid, and amikacin, these drugs continued to demonstrate less stable pooled estimates, supporting the need for cautious interpretation of their susceptibility results. There are other possibilities for the low sensitivity of PAS, CS, and MFX, because Table 4 clarifies that the absence of harmonized WHO-endorsed critical concentrations for PAS and cycloserine, together with the incompletely understood relationship between laboratory MIC values and clinically achievable moxifloxacin concentrations, may contribute to discrepancies between MIC-based and conventional critical concentration-based reference methods. For these drugs, Sensititre MYCOTB should be interpreted cautiously, ideally alongside genotypic data and clinical context, and alternative or companion agents prioritized. Several drugs included in the Sensititre MYCOTB panel, such as kanamycin, amikacin, streptomycin, ethionamide, and para-aminosalicylic acid, are currently less preferred or no longer routinely recommended in WHO MDR/RR-TB regimens. However, susceptibility testing for these agents may still be valuable in individualized DR-TB management and resource-limited settings because many included studies were conducted before recent WHO guideline updates and the adoption of newer all-oral regimens (34). Compared with line probe assays and cartridge-based molecular tests, which provide rapid detection of common resistance mutations but no MICs and may miss rare mutations or heteroresistance (24, 35, 36), BMD offers slower but more comprehensive phenotypic information. Current consensus statements emphasize that molecular and phenotypic DST are complementary and that quantitative methods, such as BMD, are needed to refine genotype–phenotype–outcome correlations and support precision dosing (23, 24, 32).

Even though bedaquiline and linezolid were not included in our analysis, recent multi-country studies show that BMD can be standardized for these medications using verified ECOFFs and quality-control ranges, enabling surveillance for new resistance in MDR/XDR programs (9, 10, 28, 37, 38). Integrating such extended BMD panels with WGS will be critical as BPaLM/BPaL regimens scale up (10, 24, 37, 38).

Methodological strengths of this review include comprehensive searching, focus on MDR/pre-XDR-enriched populations, use of bivariate/HSROC modeling, and GRADE assessment, which showed high certainty for first-line drugs and fluoroquinolones. Limitations include variable reference methods, incomplete coverage of newer agents, and limited data for cycloserine and PAS. Future work should prioritize harmonized BMD protocols, PK/PD-informed breakpoints, and the inclusion of key newer drugs (bedaquiline, linezolid, delamanid, pretomanid) to maximize clinical impact (23, 27, 28, 32, 33, 39, 40).

## CONCLUSION

Sensititre MYCOTB DST for *M. tuberculosis* shows high diagnostic accuracy for rifampicin and isoniazid and good performance for second-line injectables, supporting its use to guide regimen construction for MDR and pre-XDR TB across diverse settings. Sensititre MYCOTB aligns with modern MIC-distribution-based approaches to breakpoint determination and helps interpret genotype-phenotype discordance and heteroresistance. Even though sensitivity analysis demonstrated improved pooled sensitivity estimates for moxifloxacin and para-aminosalicylic acid, except for cycloserine, other factors, such as limited resistant isolates, wide confidence intervals, persistent heterogeneity, non-harmonized WHO breakpoints, and methodological variability across studies, also contributed. Some of the problems listed here may also apply to other drugs. Therefore, susceptibility results for these agents should still be interpreted cautiously, particularly when used for critical regimen decisions. Overall, aggregated evidence indicates that Sensititre MYCOTB plates are a strong candidate platform for routine MIC-based DST in MDR and pre-XDR TB, especially when integrated with molecular testing.

## Data Availability

Data underlying this study, including a summary of findings tables with certainty of evidence assessments and the PRISMA-DTA checklist for this manuscript, are not publicly available. These materials are available from the corresponding authors, who will provide them upon a reasonable request.
